# Significant Differences in the Effects of Pine Wilt Disease Invasion on Plant Diversity in Natural and Planted Forests

**DOI:** 10.3390/insects16030295

**Published:** 2025-03-12

**Authors:** Zijing Zhang, Jixia Huang, Zhiyao Tang, Junhao Zhao, Xiumei Mo

**Affiliations:** 1 State Key Laboratory of Efficient Production of Forest Resources, Beijing Forestry University, Beijing 100083, China; sxauzijing@163.com (Z.Z.); zhaojunhao@bjfu.edu.cn (J.Z.); mxm01292022@163.com (X.M.); 2 Academy of Plateau Science and Sustainability, People’s Government of Qinghai Province & Beijing Normal University, Xining 810008, China; 3 Institute of Ecology, College of Urban and Environmental Science, Peking University, Beijing 100871, China; zytang@urban.pku.edu.cn

**Keywords:** pine wilt disease, natural/planted forests, plant diversity, lost speed, resilience, significant differences

## Abstract

Plants, as producers in ecosystems, are an integral part of biodiversity in terms of their species diversity. Plant diversity not only enhances the quality of ecosystem services, but also provides habitat for a wide range of plants and animals. The invasion of pine wilt disease (PWD) has posed a significant threat to plant diversity in China, but it is not clear whether this threat would be significantly different in natural and planted forests. In this study, we collected a long time series of refined forest subcompartment data on PWD occurrence and plant diversity sample survey data to analyze the loss and recovery time of plant diversity in China caused by PWD invasion, especially the degree of impact on plant diversity in natural and planted forests. The results showed that after PWD invasion, the plant diversity levels of China’s national, natural, and planted forests reached a minimum in the third year of invasion, with a loss of 9.1%, 6.46%, and 9.82%, respectively, relative to the pre-invasion levels. Starting from the third year of invasion, the plant diversity levels of the three recovered gradually at different rates, among which there was a significant difference in the speed of recovery between natural forests and planted forests, which took two and three years to recover to the original level of plant diversity, respectively. This study revealed the differences in the response of plant diversity to PWD invasion between natural and planted forests and provided a theoretical basis for local governments and managers in preventing and controlling PWD and protecting plant diversity.

## Simple Summary

The diversity of species of plants, as producers in ecosystems, is an integral part of biodiversity. Plant diversity not only improves the quality of ecosystem services but also provides habitat for a wide range of plants and animals. However, in recent years, the invasion of alien species has posed a serious threat to plant diversity, especially in China. Although many studies on the effects of pine wilt disease invasion on plant diversity have been carried out both at home and abroad, there are still the problems of short time series, small study areas, and insufficient comparisons of forest types. Based on this, this study assessed the impact of pine wilt disease invasion on plant diversity across a long time series of refined forest subcompartment data on PWD occurrence and plant diversity sample survey data, especially comparing the degree of impact on plant diversity in natural and planted forests to address the shortcomings of existing studies.

## 1. Introduction

Biodiversity plays a crucial role in providing ecosystem services to human beings and in maintaining ecosystem stability [1]. However, human activities and climate change have put global biodiversity at significant risk [2,3], and the planet is losing species at an unprecedented rate, with current extinction rates at least hundreds of times higher than historical extinction rates, and the loss of biodiversity definitely exceeds the planet’s boundaries [4,5]. The loss of biodiversity has direct implications for the services and benefits it provides to humans, as well as indirect impacts on human well-being and socio-economic development.

Plant diversity, an integral part of biodiversity, serves various essential functions. It helps mitigate the greenhouse gas effect, maintains and enhances ecosystem productivity, provides habitats and food for wildlife, and improves living environments for humans [6]. Biological invasions are recognized as one of the most important drivers for the plant diversity loss globally [7]. Invasive non-native species act as “mega-disturbances”, drastically altering forest composition and ecological characteristics, thereby threatening biodiversity in both natural and planted forests worldwide [8].

Pine wilt disease (PWD) is a global forest disease caused by the pine wilt nematode (PWN, *Bursaphelenchus xylophilus* (Steiner and Buhrer, 1934)) [9]. Originating in North America and spreading through the timber trade to several countries, including Japan (1905), China (1982), Korea (1988), Portugal (1999), Spain (2008), and Africa (2009), PWD poses a serious threat to pine species [10,11,12]. In recent years, PWD has proliferated in China, causing substantial damage to the country’s ecology, natural landscapes, and socio-economy [13]. The affected areas in China have exceeded 1.51 million hectares, with over 10,404,800 diseased pine trees as of 2022 [14]. PWD has impacted ecologically important zones and landscapes, such as the Three Gorges of the Yangtze River, the Qinling-Dabashan Mountains, and has significantly affected tourism areas, such as Zhangjiajie, Mount Lushan, and Thousand Island Lake [15].

The pathogen responsible for the PWD, the PWN, feeds on shoots and disrupts the vascular system of plants, adversely affecting host health [16,17]. In addition, PWD invasion can cause extensive mortality of pine trees, leading to the destruction of understory biodiversity and posing a significant threat to forest ecosystems [18,19,20,21]. While many studies have examined the impacts of PWD on species diversity at the single stand or regional scale [22], there are still limitations in the existing studies as follows: first, most of the studies only focus on the comparison of plant diversity in a single time period after PWD invasion or in two time periods before and after the invasion, and they lack the dynamic tracking of the plant diversity after the invasion [23,24,25]; second, the natural and planted forests, as two forest types with significant differences in ecosystem services, may have different resistance and recovery potentials, but there is still a lack of relevant comparative studies [26]; and third, existing assessments mostly rely on small-sample observations, which struggle to reveal large-scale spatial heterogeneity [23,24,25,27]. These limitations seriously constrain the development of adaptive management strategies based on ecological theory. Therefore, it is necessary to compare the long-term trends of plant diversity in natural and planted forests after PWD invasion on a large scale.

In this study, we used Chinese PWD subcompartment records from 1986 to 2018, along with sample survey and remote sensing data, to evaluate the impacts of PWD invasion on plant diversity throughout China. We focus on quantifying the differential effects of PWD invasion on plant diversity in natural and planted forests, addressing the shortcomings of existing studies in terms of spatial and temporal scales and forest type comparisons, and focusing on the following three scientific questions:

1. How does plant diversity change at the three scales of national, natural, and planted forests in China following PWD invasion, and are there significant spatial variations? Studies have shown that PWD invasion can cause serious disruption of local pine resources in the short term, leading to a loss in plant diversity [13,16,21]. However, with the subsequent adoption of a series of conservation measures, such as logging of affected wood, the succession from pure forest to broad-leaved forest has been accelerated, the community structure has been improved, and the plant diversity may recover [28]. Therefore, we hypothesized that plant diversity at the three scales of national, natural, and planted forests in China would show a trend of continuous loss in the early stage and gradual recovery in the later stage. Because of the differences in the intensity of protection measures in response to PWD invasion in different areas of China [29], the study guessed that there might be significant spatial differences in the changes in plant diversity. The findings of this study will quantify the multi-scale effects of PWD invasion on plant diversity, reveal the multi-scale dynamic patterns of plant diversity after PWD invasion, and provide a scientific and theoretical basis for local governments and managers to develop effective pest management practices and plant diversity restoration measures.
2. Is there a significant difference in the loss rate of plant diversity between natural and planted forests after PWD invasion? If so, which rate of loss is faster? High species diversity in host communities may promote pest invasions by providing more ecological niches (facilitation effect), but it may also diminish invasion success because of low host dominance (dilution effect) [30]. Natural forests usually have higher plant diversity than planted forests, so we guess that PWD is less likely to occur in natural forests than in planted forests, and that the difference in invasion likelihood leads to a significant difference in the loss rate of plant diversity between the two following PWD invasions, and that the loss rate is slower in natural forests than in planted forests. If natural forests show greater resilience to invasions, priority should be given to protecting high-diversity natural forests as buffer zones against biological invasions to provide a basis for differentiated prevention and control.
3. Is there a significant difference in the rate of recovery of plant diversity between natural and planted forests in the late stage of diversity recovery after PWD invasion? If it exists, which of the two recovers faster? Natural regeneration of forests is considered to be the most effective method to restore biodiversity [31,32], and natural forests, as naturally regenerated forests, have more obvious natural regeneration compared to planted forests, so we conjecture that there is a significant difference in the rate of restoration of plant diversity in natural forests and planted forests after the PWD invasion, and the natural forests recovered faster than planted forests. The results of this study can directly guide the choice of management strategies, and if the recovery rate of natural forests is significantly superior, then the near-natural recovery mode should be preferred to reduce unnecessary afforestation inputs.

## 2. Materials and Methods

### 2.1. PWD Occurring Data

**Figure 1:**
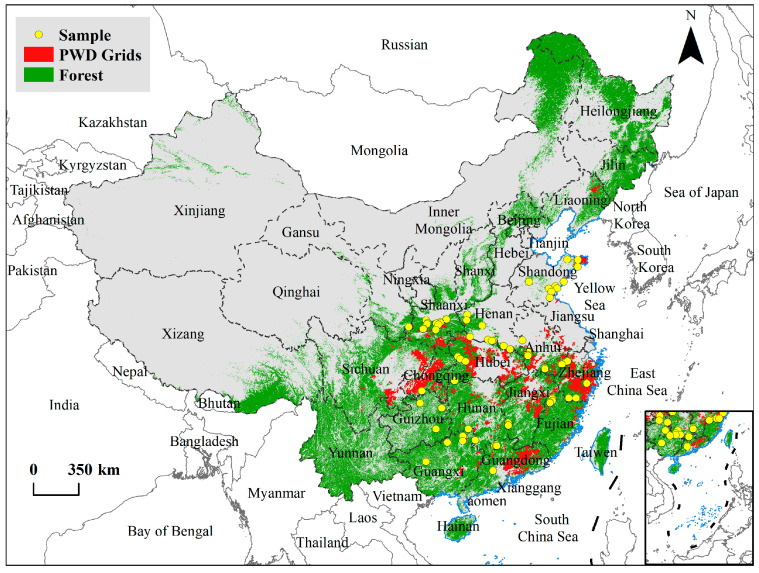
Grids for pine wilt disease 1986–2018, sample survey sites, and forest vegetation distribution in China, 2018 [33].

In China, county-level forest pest control and quarantine stations conduct an annual special survey for PWD, using the ground inspections and aerial remote sensing surveys. After sampling, isolating, and identifying the disease, the outbreak is confirmed, and detailed inspections are carried out to determine the extent of the epidemic within the jurisdiction. The information on epidemic occurrence, including the affected area, is recorded and reported to the Center for Biological Hazard Prevention and Control of the State Forestry and Grassland Administration (CBHPC-SFAA) https://www.bdpc.org.cn/ (accessed on 25 November 2023). The quarantine division of the same agency verifies the reported data. We derived the PWD-occurring data from 1986 to 2018 in China from the CBHPC-SFAA, which included 211,005 PWD records, with information on the affected area, the number of diseased and dead trees, and the origin of the forests (natural or planted forests). We used the fishnet grid tool in ArcGIS to convert the national PWD data into a 0.05° × 0.05° grid dataset under the WGS84 world geodetic coordinate system. We then defined the grids that included all classes of planted forests in the earliest year as planted forests, while other grids were natural forest grids. In total, we included 9856 grids, with 2067 as planted and 7789 grids as natural forests, respectively (Figure 1).

### 2.2. Plant Diversity Data

Based on the principles of representativeness, stability, and a convenience of plant diversity sample plot deployment, we deployed 119 forest sample plots near the PWD occurrence area surveyed during 1996–2002 and 2011–2015. Plots were selected to avoid anthropogenic and natural disturbances, such as large canopy gaps, without controlling for confounding variables such as soil composition, stand age, and climatic conditions. The size of forest plots is 600 m^2^ (20 × 30 m; 400 m^2^ in a few cases), consisting of six 10 × 10 m subplots. First, we recorded the latitude, longitude, elevation, aspect, slope, and canopy coverage for each plot. Secondly, we surveyed the species and community information of the tree layer from the whole plot and set up five 1 × 1 m quadrats at the four corners and the center of the plot for recording all the herbaceous plant species and community information occurring in the plot. Species were identified in situ by experts. For plants that could not be identified in the field, specimens were collected and identified by relevant experts. In selected sites with large elevational ranges, local air temperature starting about 1.5 m above the ground was measured at an elevation interval of 100–200 m, using HOBO microloggers (Onset Computer). Finally, we recorded data on the growth status and community characteristics of trees and herbaceous plants, together with information on longitude, latitude, elevation, and species in a community of 119 forest sample plots (Figure 1).

In this study, four types of indices, Margalef’s richness index (*MR*), Shannon’s diversity index (*H*), Simpson’s dominance index (*D*), and Pielou’s evenness index (*J*), were selected to represent plant species diversity, and no dilution method was used to take into account the differences in sampling effort (Table 1). For example, to calculate the species diversity of tree plants, we firstly counted the number of species of trees in each sample, denoted by S, and then summed the number of individuals of the species in the sample, denoted by N, and finally obtained Margalef’s richness index through the formula in Table 1; secondly, we counted the number of individuals of each species in each sample, denoted by M, and divided M by N to obtain the proportion P of each species, and finally obtained Shannon index and Simpson index in each sample; after obtaining the Shannon index, the Shannon index and Simpson index were obtained through the formula in Table 1; after obtaining the Shannon index, the Shannon index was calculated. Secondly, we counted the number of individuals of each species in each sample, denoted by M, and divided M by N to get the proportion P of each species, and then calculated the Shannon index and Simpson index of each sample by the formula in Table 1, and then calculated the Pielou evenness index by using the formula in Table 1. The calculation of the diversity of herbaceous species was the same as above. Finally, the average value of the two plant species diversity was taken as the plant species diversity of the plot.

### 2.3. Remote Sensed Data

**Figure 2:**
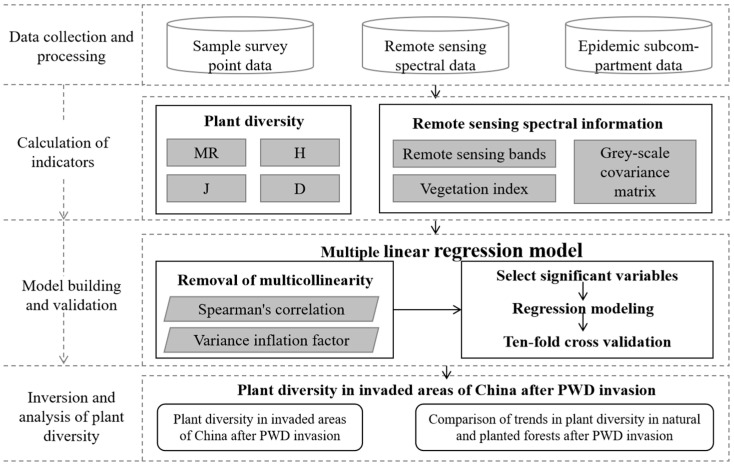
Technology roadmaps.

Remote sensing spectral information of the sampling area was extracted and modeled with four diversity indices of the sample sites as a means of inverting the plant diversity of the PWD invasion area in China. Based on the considerations of the resolution, time span, and its open-source nature, we finally selected data from the Landsat series, which provides multispectral data in the blue, green, and red visible bands, one near-infrared band, and two short-wave infrared bands, with a spatial resolution of 30 m. Specifically, in this study, we downloaded Level 2, Collection 2, and Tier 1 series datasets of Landsat 5 (1986–1999) and Landsat 7 (2000–2022) from the Google Earth Engine platform https://earthengine.google.com/ (accessed on 21 September 2023). We then analyzed data according to the following flowchart (Figure 2).

### 2.4. Relationship Between Remote Sensing Metrics and Plant Diversity

The linear regression model is widely used by researchers because of its strong data inclusiveness and intuition, which can quantitatively describe the relationship between dependent and independent variables so that researchers can clearly understand the degree of influence of different indicators on plant diversity, and, through regression analysis, hypothesis testing can be carried out to assess whether the various dependent variables have a significant impact on plant diversity and to enhance the scientific nature of the study. Therefore, in this study, three aspects of spectral information, such as the band, the vegetation index, and the textural features of the gray-level covariance matrix (GLCM), were extracted from Landsat 5 and Landsat 7 remote sensing images (Table A1) as independent variables in constructing the multiple linear regression model with plant species diversity (Table A2) [38]. The formula is as follows:

(1) $$MR=a+\sum_{i=1}^{6}b_{i}Bands_{i}+\sum_{i=1}^{4}c_{i}VIs_{i}+\sum_{i=1}^{6}d_{i}Variansce_{Bands_{i}}+\sum_{i=1}^{6}e_{i}Dissimilarity_{Bands_{i}}+\sum_{i=1}^{6}f_{i}Entropy_{Bands_{i}}$$

where *MR* represents the Margalef’s richness index; *a* is a constant term; *b_i_*, *c_i_*, *d_i_*, *e_i_*, *f_i_* denote the coefficients of band, vegetation index, and band-based texture feature variance, dissimilarity, and entropy, respectively; *Bands_i_* denotes the ith band, and the 1st–6th bands are Blue, Green, Red, NIR, SWIR1, and SWIR2, respectively; *VIs_i_* denotes the ith vegetation index, and the 1st–4th vegetation indices are NDVI, EVI, SRI, and SAVI, respectively; *Variance_Bandsi_*, *Dissimilarity_Bandsi_*, and *Entropy_Bandsi_* denote the variance, dissimilarity, and entropy of the texture features in bands 1–6, respectively. The other three diversity metrics (H, D, and J) also were subjected to the same process as Margalef’s richness index.

**Figure A1:**
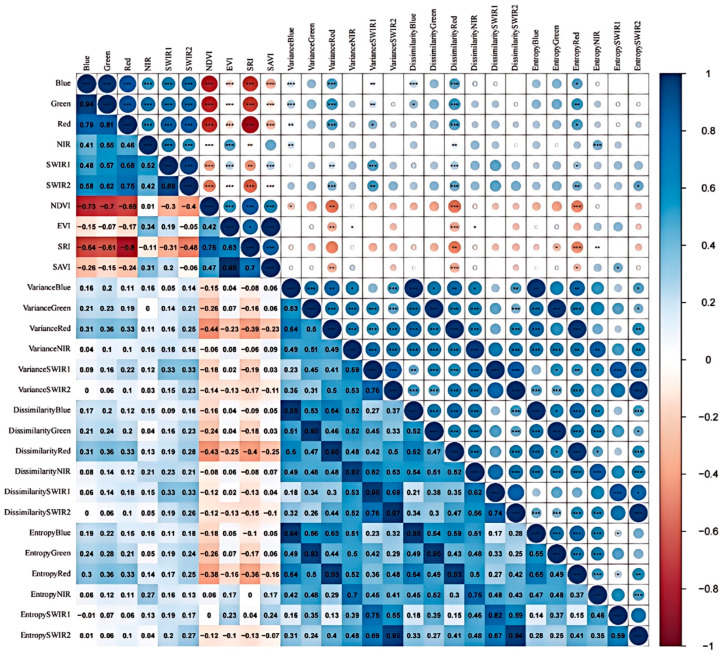
Spearman’s coefficients and *p*-values between the 28 variables. *, **, and *** indicate significant correlations at the 0.05, 0.01, and 0.001 levels, respectively.

**Figure A2:**
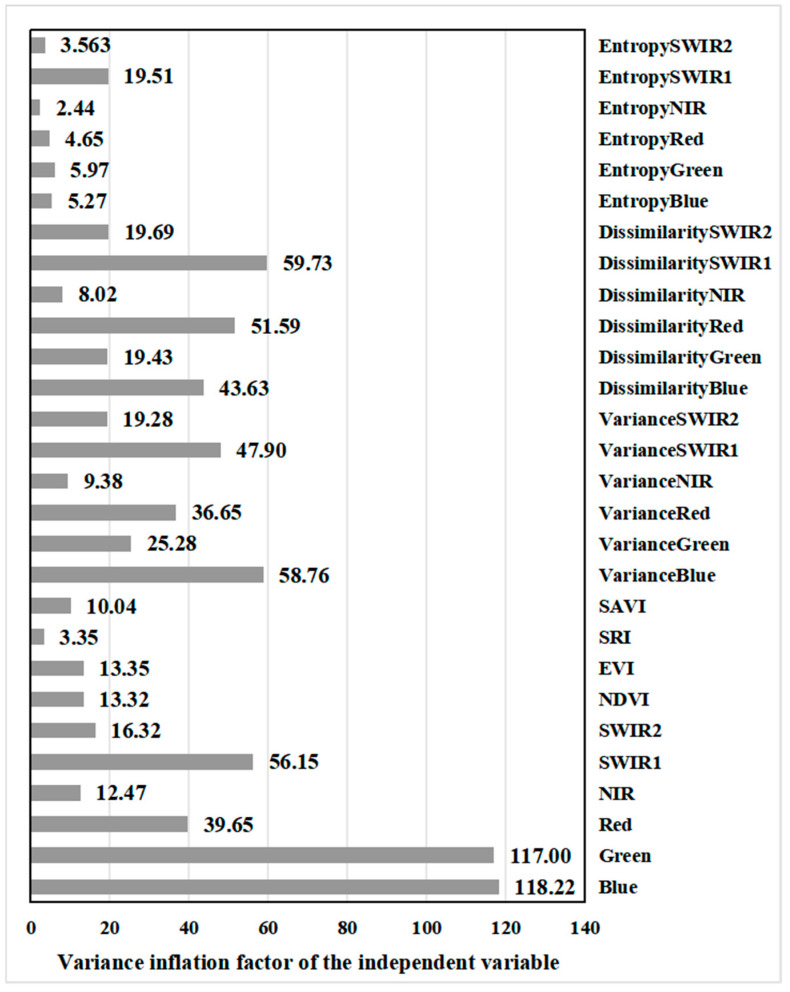
VIF values for 28 variables at three levels: remotely sensed bands (bands), vegetation indices (VIs), and gray level co-occurrence matrix (GLCM).

To account for the potential for multicollinearity among independent variables, a multicollinearity assessment was performed for each variable prior to model development. Correlation analysis and variance inflation factors are often effective methods for removing multicollinearity from variables. Correlation analysis provides a visual indication of the strength of the linear relationship between independent variables; if the correlation coefficients between two or more independent variables are very high, this indicates that there is a strong linear relationship between them, which may lead to multicollinearity; whereas the Variance Inflation Factor (VIF) provides an explicit value indicating the extent to which the variance of a particular independent variable is inflated due to the linearity of the other independent variables. The higher the value, the greater the degree to which the variable is affected by the other variables, and thus the more severe the multicollinearity problem. In general, the criteria for identifying multicollinearity were as follows: (1) a correlation coefficient greater than 0.7 with a *p*-value less than 0.05, and (2) a Variance Inflation Factor (VIF) greater than 5. In this study, Spearman’s correlation coefficients and VIFs for all variables were calculated. The results indicated the presence of multicollinearity among the variables (Figure A1 and Figure A2). To mitigate this issue, a manual variable elimination approach was employed. The elimination criterion involved removing variables with relatively high VIFs when the absolute value of the correlation coefficient (|R|) between two variables exceeded 0.7. This process resulted in the selection of 10 variables from the initial 28, which included near-infrared (NIR) and shortwave infrared (SWIR2) raw bands, two vegetation indices (SRI and SAVI), and six entropy measures derived from the raw bands. In the subsequent modeling phase, these 10 variables were used in conjunction with four types of species diversity indices. It was found that some variables did not exhibit significant relationships with the diversity indices, as indicated by *p*-values greater than 0.1. Consequently, these variables were excluded from the final model equations, ensuring that the model was free from multicollinearity and robust in its predictive capacity.

In addition, we evaluated the performance of the regression models using a tenfold cross-validation method. The evaluation metrics included the coefficient of determination (*R*^2^), root mean square error (*RMSE*), and mean absolute error (*MAE*). The formulae are as follows:

(2) $$R^{2}=1-\sum_{i=1}^{n}\left({\hat{y}}_{i}-y_{i}\right)/\sum_{i=1}^{n}\left({\bar{y}}_{i}-y_{i}\right)$$

(3) $$RMSE=\sqrt{(\sum_{i=1}^{n}{\left(y_{i}-{\hat{y}}_{i}\right)}^{2})/m}\;$$

(4) $$MAE=(\sum_{i=1}^{n}\left|{\hat{y}}_{i}-y_{i}\right|)/m$$

where $y_{i}$ denotes the actual value of the sample i, ${\hat{y}}_{i}$ denotes the predicted value of the sample i, ${\bar{y}}_{i}$ denotes the mean value of all the samples, and n denotes the number of samples.

### 2.5. Impact of PWD Invasion on the Plant Diversity

We established regression models for four plant diversity indices: Margalef’s richness index (*MR*), Shannon’s diversity index (*H*), Simpson’s dominance index (*D*), and Pielou’s evenness index (*J*), following the results of multicollinearity among variables. These models incorporated remote sensing spectral information specific to the invasive PWD in China. By inputting this spectral data into the regression models, the study was able to estimate the values of *MR*, *H*, *D*, and *J* for each year from 1986 to 2022 within the invasion area.

However, it was noted that due to the inherent limitations of the model, some grid cells exhibited species diversity indices that fell outside a reasonable range. To address this issue, we developed a combined plant diversity index (*ComDiv*) that aggregated the four diversity indices. This combined index provided a standardized measure of plant diversity for the region, with a value scale of 0 to 1. A higher value closer to 1 indicates a higher level of plant diversity in the area. This approach served to normalize the diversity indices and mitigate the impact of model errors, thereby enhancing the reliability of the study’s findings. The formula for the combined plant diversity index is shown in (5):

(5) $$\begin{array}{c} ComDiv=[(MR-MR_{\min})/(MR_{\max}-MR_{\min})+(H-H_{\min})/(H_{\max}-H_{\min})+(D-D_{\min})/(D_{\max}-D_{\min})+\\ (J-J_{\min})/(J_{\max}-J_{\min})]/4 \end{array}$$

where *ComDiv* represents the combined plant diversity index; *MR*, *H*, *D,* and *J* represent the Margalef’s richness index, the Shannon diversity index, the Simpson dominance index, and the Pielou evenness index, respectively. *MR_min_*, *H_min_*, *D_min_*, and *J_min_* denote the minimum values of the four species diversity indices in all grids and *MR_max_*, *H_max_*, *D_max_*, and *J_max_* denote the maximum values of the four species diversity indices in all grids, respectively.

We further investigated the effects of PWD invasion on plant diversity in China, examining both trends and spatial variation. Firstly, we generated the combined plant diversity of all grids under different PWD invasion times based on the first year of the grids and the year-by-year combined plant diversity over the last four decades. Secondly, we separately averaged the combined plant diversity of all grids under each invasion time and analyzed the trend of diversity change in China based on these average values. Finally, based on the plant diversity level of the grids in the first year of PWD invasion, we calculated the changes in plant diversity for all grids in the following years, thereby spatially analyzing whether there are obvious regional differences in the changes in plant diversity.

To illuminate the contrasting effects of PWD on natural and planted forests, we classified the forest origins of all grids based on the forest type data from the PWD subgroups at the national scale. Drawing upon the analytical framework established for studying China’s plant diversity trends, we then conducted a comparative analysis of biodiversity shifts in these two forest categories.

## 3. Results

### 3.1. Relationship Between Remote Sensing Spectral Features and Plant Species Diversity

The performance of the four regression models has some generalization ability (Table A3). Although the R^2^ of the four regression models is low, the values of RMSE and MAE are small, in which, the RMSEs of the four regression models *MR*, *H*, *J*, and *D* are 2.081, 0.496, 0.265, and 0.238, and the MAEs are 1.644, 0.374, 0.207, 0.172, which indicates that the gap between the predicted and the real values is small, and the model has a high prediction accuracy.

**Figure 3:**
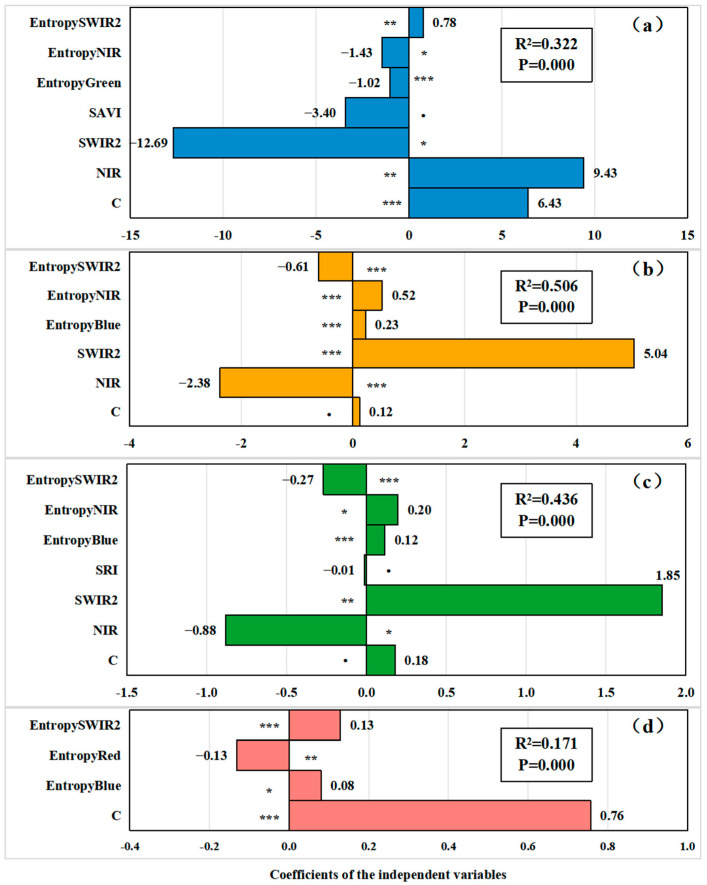
Figure (**a**–**d**) represent the regression results of the *MR*, *H*, *J*, and *D* plant species diversity index and remote sensing-related variables, respectively. *, ** and *** indicate significant correlations at the 0.05, 0.01, and 0.001 levels, respectively.

The regression models showed that the entropy texture features of the gray-level covariance matrix were significantly correlated with the four species diversity indices (Figure 3). Specifically, the texture feature entropy calculated from all green (EntropyGreen), near-infrared (EntropyNIR), and short-wave infrared (SWIR2) (EntropySWIR2) bands significantly correlated with *MR* (*p* < 0.05 for all, Figure 3a); EntropyBlue, EntropyNIR, and EntropySWIR2 were significant for H (*p* < 0.001 for all, Figure 3b) and J (*p* < 0.05 for all, Figure 3c); and EntropySWIR2, EntropyRed, and EntropyBlue were significant for D (*p* < 0.05 for all, Figure 3d).

### 3.2. Changes of Plant Diversity with PWD Invasion

**Figure 4:**
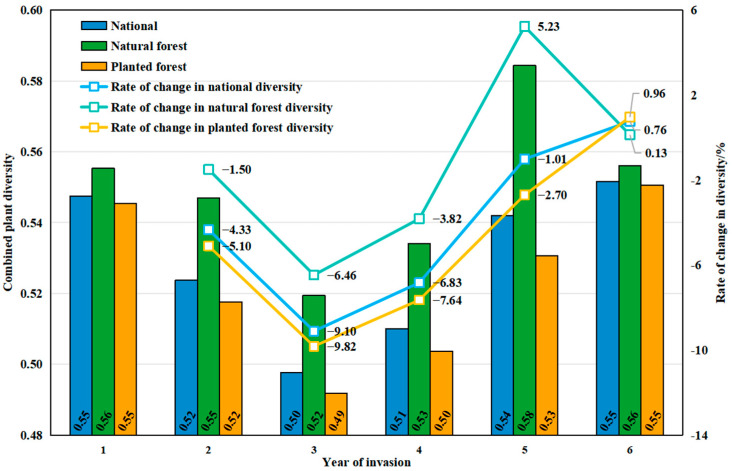
After the PWD invasion, Chinese natural and planted forests’ year-by-year average *ComDiv* and rate of change (%). (The rate of change is calculated based on the plant diversity level in the first year of invasion).

On average, the *ComDiv* declined from approximately 0.55 at the onset of PWD invasion to 0.52 in the first subsequent year, then to 0.50 in the second year, reaching its lowest point (0.50) in the third year. Subsequently, the average *ComDiv* increased and returned to 0.55 by the sixth year after the invasion (Figure 4).

**Figure 5:**
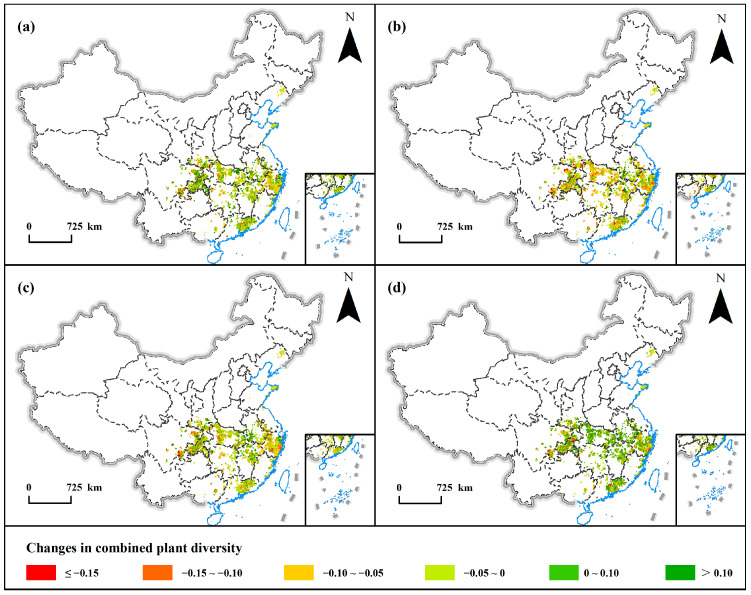
(**a**–**d**) represent the distribution of changes in the *ComDiv* in China after one year, two years, three years, and four years of PWD invasion, respectively.

The spatial distribution of changes in the *ComDiv* after PWD invasion shows that, one year after the emergence of PWD, nearly 70% of the areas in the country showed a loss of *ComDiv* (Table A4). Compared to the initial invasion, there was an overall loss of 4.33% of *ComDiv* (Figure 4), with significant diversity fluctuations in Sichuan, Chongqing, Shaanxi, Hubei, Zhejiang, and Guangdong, where some areas lost more than 0.15 of *ComDiv* (Figure 5a). By the second year post-occurrence, a more pronounced loss of diversity was observed nationwide, with the *ComDiv* losses occurring in more than 80% of the country (Table A4). The national *ComDiv* lost roughly 9.1% compared to levels at the time of the initial invasion (Figure 4). At this juncture, spatial variations in the *ComDiv* changes across provinces were conspicuous, with particularly large losses in Sichuan, Chongqing, and Zhejiang, while certain areas of Anhui and Jiangxi Provinces demonstrated recoveries surpassing 0.1 (Figure 5b). Three years after the event, the national *ComDiv* began to recover, with the rate of change decreasing from −9.1% to −6.83% (Figure 4). Most significant losses were concentrated in the Sichuan and Zhejiang Provinces, while fluctuations in the *ComDiv* elsewhere generally hovered around 0.1 (Figure 5c). By the fourth year post-occurrence, the national *ComDiv* had essentially recovered to pre-invasion levels, with over 90% of regions exhibiting diversity changes of less than 0.1 (Table A3). Notably, spatial diversity disparities persisted, notably evident in the regions of Sichuan, Chongqing, and Zhejiang (Figure 5d).

### 3.3. Different Effects of PWD Invasion on Plant Diversity Between Natural and Planted Forests

The PWD grid delineation based on the forest origin field of the PWD subcompartment revealed that approximately 79% of observed PWD cases were in planted forests, and the risk of pine wilt disease in planted forests appeared to be four times higher than in natural forests.

In average, the average *ComDiv* for both natural and planted forests declined, respectively, from approximately 0.56 and 0.55 at the onset of PWD invasion to a minimum of 0.52 and 0.49 after two years. Subsequently, the *ComDiv* for natural and planted forests then recovered to 0.58 and 0.55 by the fifth and the sixth year after the invasion, respectively (Figure 4).

In general, the difference between the average *ComDiv* of natural and planted forests recovered from approximately 0.01 at the beginning of the invasion, rising to 0.03 by the second year of the invasion. This difference remained at 0.03 for the next two years. Subsequently, the difference between the *ComDiv* of natural and planted forests changed dramatically, first increasing to 0.5 by the fifth year after the invasion and then decreasing to 0 by the sixth year after the invasion (Figure 4).

Trends in the average *ComDiv* showed that the *ComDiv* of both natural and planted forests showed a continuous loss in the first three years of the invasion (Figure 4). Specifically, in the second year of PWD invasion, the *ComDiv* in natural forests lost 1.5%, while in planted forests it lost 5.1%, signifying that the loss rate in planted forests was about 3.5 times that of natural forests. In the third year of PWD invasion, both forest types reached their lowest diversity values of 0.52 and 0.49, with the loss rate in planted forests approximately 1.5 times that in natural forests. In the following three years, the *ComDiv* of natural and planted forests began to recover gradually (Figure 4). Specifically, natural forests required two years to surpass their plant diversity level by the fifth year of PWD invasion, while planted forests took three years to return to their original diversity level of 0.55 by the sixth year of PWD invasion.

## 4. Discussion

### 4.1. Continued Short-Term Loss and Gradual Recovery of Plant Diversity in China After PWD Invasion

The study’s findings indicated that after PWD invasion, plant diversity in China continued to be lost in the short term, followed by gradual recovery. Firstly, PWD primarily targeted pine trees, especially those with lower resistance or susceptibility to infection. As pine trees are the main hosts of the disease, their substantial mortality resulted in a reduction in the number and species of pine trees in pine-dominated plant communities [13]. Secondly, the invasion of PWD led to shifts in competitive relationships within plant communities. With the demise of pine trees, other tree species and plants vied for the habitat resources previously utilized by the pines, such as light, water, and nutrients. This competitive dynamic could lead to an increase in the number of certain plant populations while potentially decreasing others or causing their exclusion. Additionally, PWD invasion often triggered novel and complex species interactions within and among trophic levels [39,40,41], with potential socio-economic consequences such as the emergence of new human diseases and outbreaks of agricultural and forest pests [42,43]. Previous studies have shown that PWD invasion can cause substantial damage to pine forests and ecosystems in the short term, leading to a loss of plant diversity [13,16,21].

The effects of PWD on plant community structure and species composition may result in ecological niche redistribution. Some native plants have the ability to adapt to new habitat conditions and competitive relationships, gradually filling the voids left by pine mortality [44,45]. This process could lead to a gradual increase in native plant diversity over time. Moreover, the temporal development of biodiversity after disturbances is often influenced by human interventions [46]. Conservation measures, such as logging infected trees and applying elicitors, were employed post-PWD invasion to facilitate the transition from pure forests to broad-leaved forests. These transitions have effectively contributed to a restoration in plant species richness and diversity, enhancing community structure and fostering the development of more stable ecosystems.

### 4.2. Planted Forests Lose Plant Diversity Faster than Natural Forests After PWD Invasion

Plant diversity loss is the reduction or disappearance of the number of plant species, genetic diversity, or ecosystem functions in a given area. The study found that approximately 79% of the observed PWD cases were in planted forests and that planted forests were more adversely affected by PWD invasion in comparison with natural forests, with a significantly greater loss of diversity than natural forests. This significant difference can be explained in depth by the following ecological theoretical frameworks and mechanisms.

First, the Disturbance–Invasion Hypothesis states that disturbed habitats (e.g., planted forests) are more vulnerable to invasion because disruptions create gaps in the ecological niche and alter the allocation of resources (e.g., light, nutrients), thereby weakening the resistance of ecosystems [44,45]. Frequent anthropogenic disturbances (e.g., logging, monoculture planting) in planted forests significantly increase the risk of PWD invasion [46,47].

Second, the Diversity–Stability Hypothesis suggests that high species diversity in natural forests enhances resistance through the following mechanisms: (1) The Dilution Effect: non-host plants (e.g., broadleaf trees) reduce the efficiency of PWD transmission by isolating the host (pine plants) [48,49,50]. (2) The Enemy Release Hypothesis: Natural forests with high diversity support more natural enemies (e.g., parasitic wasps, predatory beetles), which inhibit the expansion of pine nematode populations [51]. (3) The Resource Partitioning Theory: diverse plant communities reduce the concentrated supply of resources from a single host through ecological niche differentiation, reducing the adaptability of pests [50].

However, the Invasion Paradox suggests that while high diversity generally inhibits invasion, in some cases diverse communities may promote specific invasive species by providing richer microhabitats [46]. This apparently contradictory conclusion, described as the Invasion Paradox, is partially reconciled by the conclusion that the native/exotic species richness relationship is driven by a variety of ecological factors that vary in importance at different spatial scales [52].

### 4.3. Plant Diversity in Natural Forests Recovered More Rapidly than in Planted Forests After PWD Invasion

Resilience is the ability of an ecosystem to maintain its basic structure and functioning and to return to its original state after a disturbance (e.g., natural disaster, climate change, or human activity). In this study, it was found that natural forests were more resilient than planted forests in terms of plant diversity recovery after PWD invasion, with natural forests taking only two years to recover above the original level of plant diversity, whereas planted forests took three years to recover basically to the original level. Natural regeneration of forests is often considered to be the most effective method for restoring biodiversity [31,32]. Natural forests, as naturally regenerating forests, have species composition stability [53]. Natural forests demonstrate a smaller change in species composition after PWD invasion. For example, the seed bank of the original companion species (e.g., cycads and bitterbrush) of the natural pine forest in the Zhoushan Islands, Zhejiang Province, sprouted rapidly after invasion, maintaining the diversity of the tree layer [23]. In addition, the restoration of natural forests conforms to the succession path of ‘initial community—competitive exclusion—stable community’ [54,55,56]. In contrast, planted forests are mainly forest ecosystems established through artificial activities, such as seeding, cultivation, and nurturing, with a low degree of natural regeneration, and their restoration faces significant constraints, which are limited by the stochastic nature of species dispersal and the need to rely on artificial interventions (e.g., the replanting of native tree species) to break the ‘ecological niche lock’ [50,57]. In addition, the restoration process of planted forests has been shown to be a major challenge. In addition, soil microbial communities recover slowly during plantation forest restoration, which further slows the re-establishment of plant diversity [17].

### 4.4. Limitations

This study has certain limitations. Firstly, the change in plant diversity is influenced by various factors such as meteorological, anthropogenic, and topographic ones, as well as the degree of invasion. This study merely focused on the change and loss of plant diversity in China under the influence of PWD invasion, without taking into account the contribution of other factors. Further research is necessary to explore the extent to which the change in diversity results from PWD invasion and the relative significance of other factors. Secondly, due to the constraints of Landsat data availability, this study did not consider the future impact of PWD invasion on plant diversity in China, which demands further investigation.

## 5. Conclusions

This study quantifies the multi-scale effects of PWD invasion on plant diversity, reveals the multi-scale dynamic patterns of plant diversity after PWD invasion, explains the resistance mechanisms of diversity, and provides key evidence for the ecosystem restoration objectives of the Kunming–Montreal Framework. The results suggest that we can optimize the management of plantation forests by introducing non-host species into plantation forests and mimicking the mixed structure of natural forests through measures, and, at the same time, we can protect and restore natural forests by establishing natural forest protection zones in areas of high PWD prevalence and limiting anthropogenic disturbances. In terms of invasion prevention and control techniques, we can inhibit the spread of PWD by introducing natural enemies of the pine ink skink. In conclusion, our study effectively remedies the shortcomings of current studies on the impact of PWD invasion on plant diversity in terms of spatial and temporal scales and the comparison of forest types and provides a theoretical foundation for local governments and forest managers for developing effective pest management measures, as well as plant diversity conservation strategies.

## Figures and Tables

**Table 1:** Plant diversity indices used in the study and their equations.

Species Diversity Index	Equation	Reference
Margalef’s richness index	MR=(S−1)/lnN	[34]
Shannon index	$H=-\sum P_{i}\ln P_{i}$	[35]
Simpson index	$D=1-\sum P_{i}^{2}$	[36]
Pielou evenness index	J=H/lnS	[37]

where *S* is the number of species in each quadrant, *N* is the sum of the individuals of all species, and *P_i_* is the proportion of the individuals of this species in the total number of individuals.

## Data Availability

Data are contained within the article.

## Abbreviations

The following abbreviations are used in this manuscript:

PWD	pine wilt disease
PWN	pine wilt nematode
*MR*	Margalef’s richness index
*H*	Shannon’s diversity index
*D*	Simpson’s dominance index
*J*	Pielou’s evenness index
GLCM	Gray-level covariance matrix
VIF	Variance Inflation Factor
R^2^	the coefficient of determination
RMSE	root mean square error
MAE	mean absolute error
*ComDiv*	combined plant diversity index

## Appendix A

**Table A1:** List of spectral datasets used as predictor variables in the models.

Variables		Equation/Spectral Bands
Vegetation indices	Normalized Difference Vegetation Index (NDVI)	=(NIR − RED)/(NIR + RED)
	Enhanced Vegetation Index (EVI)	=2.5 × (NIR − RED)/(NIR + 6.0 × RED − 7.5 × BLUE + 1.0)
	Simple Ratio Index (SRI)	=NIR/RED
	Soil Adjusted Vegetation index (SAVI)	=(NIR − RED)/(NIR + RED + L) × (1 + L), L = 0.5
Landsat spectral bands	Blue band	0.45–0.52 µm
	Green band	0.52–0.60 µm
	Red band	0.63–0.69 µm
	Near-infrared band	0.76–0.90 µm
	Shortwave infrared band-1	1.55–1.75 µm
	Shortwave infrared band-2	2.08–2.35 µm
Gray-level co-occurrence matrix textural layers	Variance	$=\sum_{i=0}^{G-1}\sum_{i=0}^{G-1}{\left(i-\mu \right)}^{2}P(i,j)$
	Dissimilarity	$=\sum_{i,j=0}^{N-1}Pi,j|i-j|$
	Entropy	$=-\sum_{i=0}^{G-1}\sum_{i=0}^{G-1}P(i,j)\times log(P(i,j))$

where L represents a constant soil adjustment factor; G represents number of gray levels used; N represents the number of distinct gray levels in the quantized image; μ represents the mean value of P; P(i,j) represent the (i,j)th entry in the normalized gray-tone spatial-dependence matrix, = P(i,j)/R; and R represents a normalizing factor. The information in the Equation/Spectral bands column is taken from the literature [38].

**Table A2:** Independent variables involved in multiple linear regression modeling.

No.	Independent Variable	No.	Independent Variable
1	Blue	15	VarianceSWIR1
2	Green	16	VarianceSWIR2
3	Red	17	DissimilarityBlue
4	NIR	18	DissimilarityGreen
5	SWIR1	19	DissimilarityRed
6	SWIR2	20	DissimilarityNIR
7	NDVI	21	DissimilaritySWIR1
8	EVI	22	DissimilaritySWIR2
9	SRI	23	EntropyBlue
10	SAVI	24	EntropyGreen
11	VarianceBlue	25	EntropyRed
12	VarianceGreen	26	EntropyNIR
13	VarianceRed	27	EntropySWIR1
14	VarianceNIR	28	EntropySWIR2

**Table A3:** Cross-validation results for four regression models.

	RMSE	R^2^	MAE
*MR*	2.081	0.300	1.644
*H*	0.496	0.493	0.374
*J*	0.265	0.441	0.207
*D*	0.238	0.239	0.172

**Table A4:** Statistics on the number and percentage of grids with the different *ComDiv* changes throughout China after one year, two years, three years, and four years of PWD invasion.

Diversity Change	Duration of Invasion			
	One Year	Two Years	Three Years	Four Years
≤0.15	214 (2.17%)	552 (5.6%)	382 (3.88%)	258 (2.62%)
−0.15~−0.10	643 (6.52%)	1214 (12.32%)	962 (9.76%)	500 (5.07%)
−0.10~−0.05	2066 (20.96%)	2967 (30.1%)	2473 (25.09%)	1343 (13.63%)
−0.05~0	3950 (40.08%)	3487 (35.38%)	3690 (37.44%)	3102 (31.47%)
0~0.1	2723 (27.63%)	1464 (14.85%)	2181 (22.13%)	4161 (42.22%)
>0.1	260 (2.64%)	172 (1.75%)	168 (1.7%)	492 (4.99%)
